# Cross-herpesvirus immunity of the cytomegalovirus gB/MF59 vaccine response

**DOI:** 10.1038/s41541-025-01315-6

**Published:** 2025-12-05

**Authors:** A. Lankina, A. Hargreaves, W. T. Lui, B. Kropff, A. Wei, J. Breuer, R. E. White, M. Thomas, P. D. Griffiths, M. B. Reeves

**Affiliations:** 1 Institute of Immunity and Transplantation, University College London, London, UK; 2 Virologisches Institut, Klinische und Molekulare Virologie, Friedrich-Alexander-Universität Erlangen-Nürnberg, Erlangen, Germany; 3 Great Ormond Street Institute of Child Health, University College London, London, UK; 4 Department of Infectious Disease, Imperial College London, London, UK

**Keywords:** Computational biology and bioinformatics, Immunology, Microbiology

## Abstract

Vaccination against human cytomegalovirus (HCMV) to protect transplant recipients and prevent congenital infection remains highest priority. Follow-up analyses of a vaccine directed against the fusion protein glycoprotein B (gB/MF59) identified a vaccine-specific response (AD-6) that correlated with protection. Subsequently, it was demonstrated that AD-6 antibodies are anti-viral by preventing cell-associated spread. Here we now demonstrate AD-6 antibodies limit HCMV reactivation – an event critical for pathogenesis via hematogenic spread in vivo. To better understand the AD-6 immunogen, we use structural homology to identify putative AD-6 regions in related herpesviruses and show, despite limited sequence similarity, they share key physico-chemical properties. Of note was that AD-6 mapped to a region under high molecular frustration within gB – arguing AD-6 antibodies inhibit gB function by targeting activity dependent on conserved conformational changes. Consistent with structural conformation being crucial, we observe that both rabbit and human HCMV AD-6 antibodies recognise other herpesvirus AD-6s and that AD-6 antibodies are potently antiviral against HSV-1. Thus, a combinatorial in silico, biochemical and immunological approach reveals conformational epitopes within AD-6 are critical components of the gB/MF59 vaccine, represent crucial conserved elements of AD-6 in gB structure and function which makes it an attractive target of multiple herpesviruses.

## Introduction

Human cytomegalovirus (HCMV) is the leading viral cause of congenital disease and remains an important complication in immune-suppressed organ transplant recipients^1–4^. It is the growing costs of managing HCMV end-organ disease and associated conditions that underpin the high-priority status of HCMV for the development of a vaccine^5,6^.

Despite a concerted effort using vaccines of multiple modalities, none has achieved the protection required for licensure^7^. To date, the best performing vaccine is based on a recombinant modified form of glycoprotein B (gB)—the fusion protein essential for HCMV entry^8,9^. Indeed, it is noteworthy that despite the inclusion of gB in multiple HCMV vaccines that have been tested, it is the gB/MF59 used alone that has given the best results so far^10–12^. Consequently, understanding the mechanistic basis for this clinically relevant protection has been of major interest.

Recently, we identified that the gB/MF59 vaccine induced antibody responses directed against a novel antigen domain (AD-6) within gB that were rarely detected in naturally infected people^13^. This suggests that gB/MF59 presents certain immunological epitopes (including AD-6) more readily than natural gB and that these atypical responses are potently anti-viral in vaccinated transplant patients. For AD-6, we hypothesise this is via blockade of cell-to-cell spread of HCMV, which is considered the main route of HCMV dissemination in vivo.

A detailed understanding of the functional, immunological and structural basis of AD-6 is critical for defining (and refining), this important response to the gB/MF59 vaccine. Here we first show that AD-6 antibodies potently block HCMV reactivation in vitro—a major source of HCMV pathogenesis in vivo via hematogenic spread^14,15^. Next, using a structural approach, we identify AD-6 within other human herpesviruses (HHV) and demonstrate that, despite very limited sequence homology, antibodies directed against HCMV AD-6 cross-react and control other HHV in a similar way. By comparing a polyclonal AD-6 antibody (pAb) with a monoclonal AD-6 antibody (mAb), we demonstrate that this cross-reactivity is dependent on conformation-specific antibodies and identify regions within AD-6 critical for this response. Furthermore, we demonstrate that human vaccine derived AD-6 antibodies recognise AD-6 in herpes simplex virus 1 (HSV-1), further supporting conformation-specific antibodies as crucial for the broad control seen in vivo. Finally, we demonstrate that the structural conformation of AD-6 across HHV imparts specific physicochemical properties important likely important for gB function. Specifically, we demonstrate AD-6 maps onto a region of gB under high molecular frustration (MF)- an approach that identifies regions of proteins energetically unstable which play crucial roles in the function of dynamic proteins that undergo topological changes like gB fusion protein. Taken together, these data demonstrate how AD-6 is an important immunological target against HCMV, define a structural and biochemical basis for this, demonstrate how the region provides cross-protective immunity, and discuss how this could be an important general strategy for the control of other HHV by humoral immunity.

## Results

### AD-6 antibodies block HCMV reactivation

**Fig. 1:**
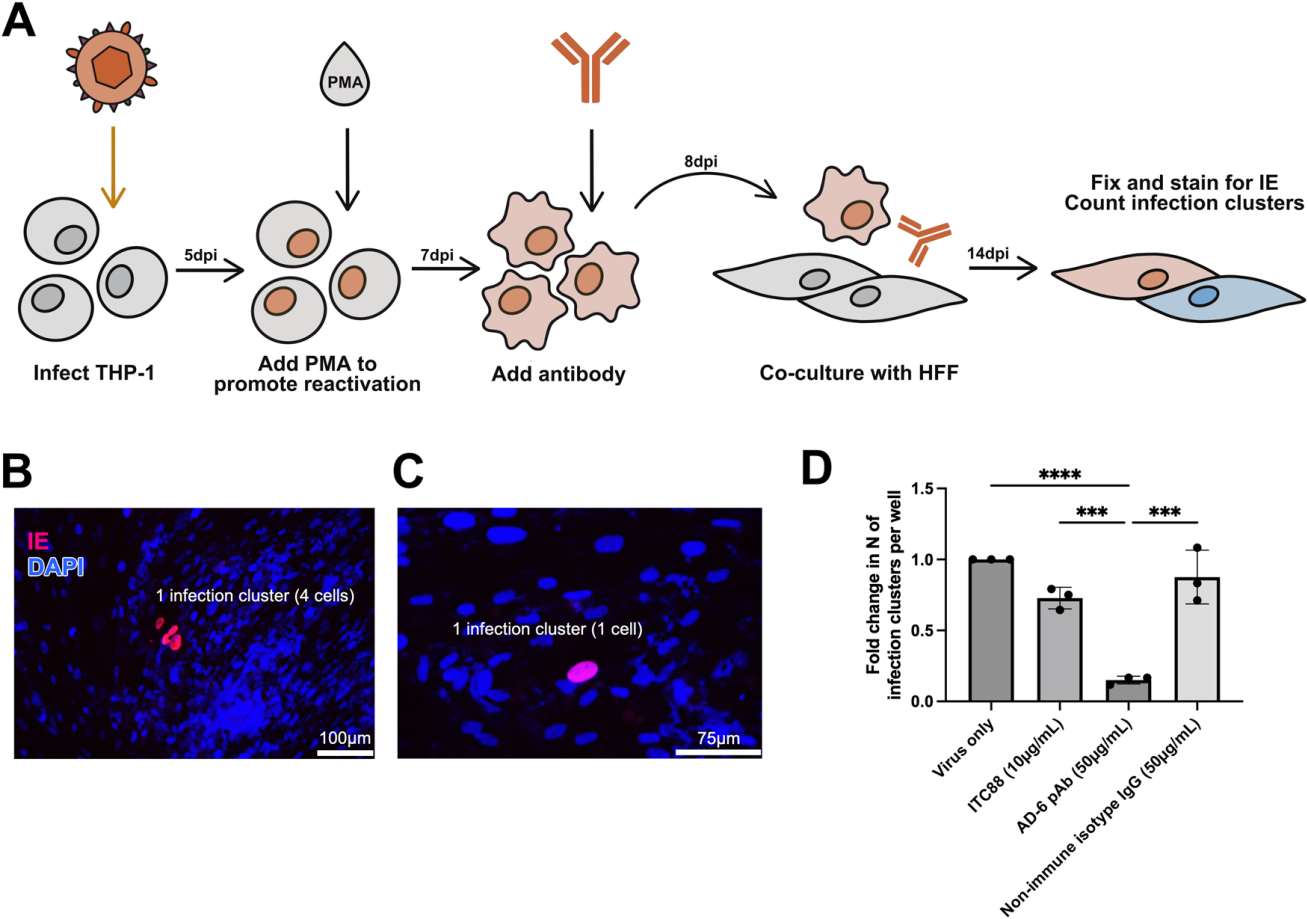
AD-6 antibody inhibits HCMV reactivation. **A** Monocytic THP-1 cells were first infected with HCMV and then, after 5 days, incubated with PMA to promote reactivation. Two days post PMA stimulation, THP-1 cells were incubated with AD-6 antibody, AD-2 antibody (ITC88) or rabbit isotype control overnight and then co-cultured with HFFs. After 7 days of co-culture, infectious foci/clusters in HFFs (reactivation) were visualised by staining for IE as a marker of infection. Clusters (**B**) or single cell foci (**C**) were scored as infectious clusters across all conditions. **D** Quantification was performed on biological replicates and the number of infectious clusters was scored per well and then expressed relative to the virus-only control. Statistical analysis was performed using the Kruskal–Wallis test with Dunn’s multiple comparisons test. Data represented as mean ± SD. *p < 0.05; **p < 0.01; ***p < 0.001; ****p < 0.0001, non-significant comparisons not shown. **A** was created by Anastasia Lankina using a ProCreate Art Package.

To better appreciate the potential clinical importance of AD-6 antibody responses, we first investigated their ability to control HCMV reactivation—a major source of viremia and hematogenic spread in vivo. To do this, we measured the impact of antibodies on the ability of HCMV to disseminate from reactivating THP-1 monocyte-like cells into fibroblasts—to model the first, critical events of clinical reactivation (Fig. 1A–C). First, we assessed the impact of an AD-6 polyclonal antibody at a concentration we have previously demonstrated to block cell-associated spread of HCMV but with no capacity to neutralise cell-free virus^13^. Interestingly, the AD-6 antibody clearly reduced the number of infectious foci in the HFF monolayer co-cultured with reactivating THP-1 macrophages (Fig. 1D). Importantly, no effect was observed with a rabbit polyclonal isotype control (Fig. 1D). Next, we wished to understand the contribution of cell-free virus spread in the same assay. To do this, we utilised the potent neutralising antibody, ITC88, which targets AD-2 of gB^16^. We chose a concentration of ITC88 that we have previously demonstrated to specifically inhibit cell-free but not cell-associated viral spread in HFF cells^17^. The data show that ITC88 had no effect on the formation of infectious foci in the HFF co-cultures (Fig. 1D). Taken together, these data are consistent with the concept that the main route of reactivation from myeloid cells is via cell-to-cell transmission and that AD-6 antibodies potently block it.

### Putative AD-6 regions share physiochemical properties across gB despite a lack of sequence identity

The gB protein is an essential fusogenic protein that is functionally conserved across the Herpesviridae family, explaining our interest in the potential immunological importance of AD-6 in gB of other HHV^18–20^. To investigate this in more detail, we first needed to identify the putative AD-6 in other HHV, which was not possible using standard sequence alignments due to low percentage identity (Supplementary Table 1). In contrast to HHV, a sequence-based approach was applicable in identifying AD-6 analogues in animal CMV gB sequences, suggesting sequence divergence had occurred early in the HV lineage (Supplementary Fig. 1).

Thus, we employed an alternative approach utilising the high level of structural homology that has been well characterised through the study of available post-fusion structures of HHV gB^20^, which we confirmed here using FATCAT analyses (Supplementary Table 3; Supplementary Fig. 3).

**Fig. 2:**
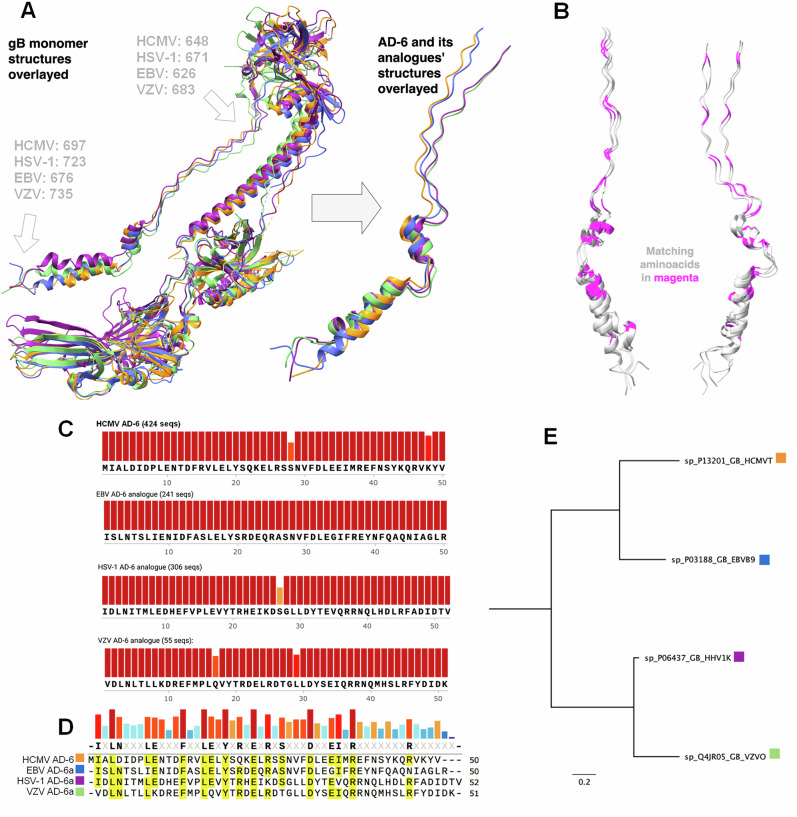
Conservation of structural and immunological motifs in glycoprotein B across the herpesvirus family. **A** Four gB structures (PDB 7KDD (HCMV), 3FVC (EBV), 3NWF (HSV-1), 6VLK (VZV)) were overlayed to identify putative amino acid coordinates of AD-6-equivalent fragments in HSV-1, EBV, and VZV. **B** Overlay of AD-6 regions of HHV gB with same or similar amino acids (magenta) on the planar AD-6 structure (left) and the opposing 180° plane (right). **C** Using the coordinates from (**A**), the sequence of AD-6 analogues was determined using representative gB sequences: UniProt P13201 (HCMV), P03188 (EBV), P06437 (HSV-1), Q4JR05 (VZV). Protein sequences for the similarity analysis are from GenBank. **D** Alignment of AD-6 sequences across HHV gB. **E** Phylogenetic analysis of AD-6 across HHV gB.

Specifically, we aligned structures of gB from HCMV, HSV-1, Varicella Zoster Virus (VZV), and Epstein-Barr Virus (EBV) and using the structural information, we identified the putative amino acid coordinates of the corresponding AD-6 on other HHV (Fig. 2A), which allowed us to interrogate properties of AD-6 comparatively across the herpesviruses (Fig. 2B–E).

Interestingly, just like HCMV AD-6, the analogous AD-6 sequences are highly conserved within their virus family (Fig. 2C) but yet again present a low level of amino acid similarity when compared with each other (Fig. 2D). Furthermore, a phylogenetic analysis (Fig. 2E) demonstrates the higher level of similarity among alphaherpesviruses (HSV-1 and VZV), and them branching out from the beta (HCMV) and gamma (EBV) subfamilies, which is concordant with our understanding of herpesviral evolution^21^. Perhaps most intriguing was that, despite the overall lack of sequence similarity, the identification of AD-6 in this way revealed a pattern whereby the same or similar amino acid is presented at regular intervals of 3 or 4 amino acids, concordant with the length of a turn of an alpha helix (3.6aa)^22^. Furthermore, when transposed back onto the rigid structure (Fig. 2B), conserved amino acids tend to be presented on the same plane of view, suggesting that conformationally conserved epitopes could exist across the four HHV AD-6 sequences analysed. Indeed, this discord between low amino acid sequence conservation but highly related structural information was also evident from hydrophobicity and electrostatic profiles, which demonstrated that the physicochemical properties of the amino acids were highly similar for all AD-6 sequences analysed (Supplementary Fig. 2). Finally, to complement the in silico structural studies, we generated predicted immunogenicity plots of all HHV gB which were revealed to be broadly similar across the gB of HHV (Supplementary Fig. 4A). Importantly, the AD-6 region was predicted to be immunogenic with 3 distinct peaks within HCMV AD-6, where the second peak is the largest one and broadly maps onto an alpha-helical region of the domain (Supplementary Fig. 4B). Similarly, in other HHV gB AD-6, an immunogenicity peak can also be observed in the middle of the domain. Thus, taken together, these data raised the hypothesis that this conserved structural region of gB could have important conformational epitopes from an immunological point of view.

### A HCMV AD-6 polyclonal antibody displays anti-viral activity against other herpesviruses

**Fig. 3:**
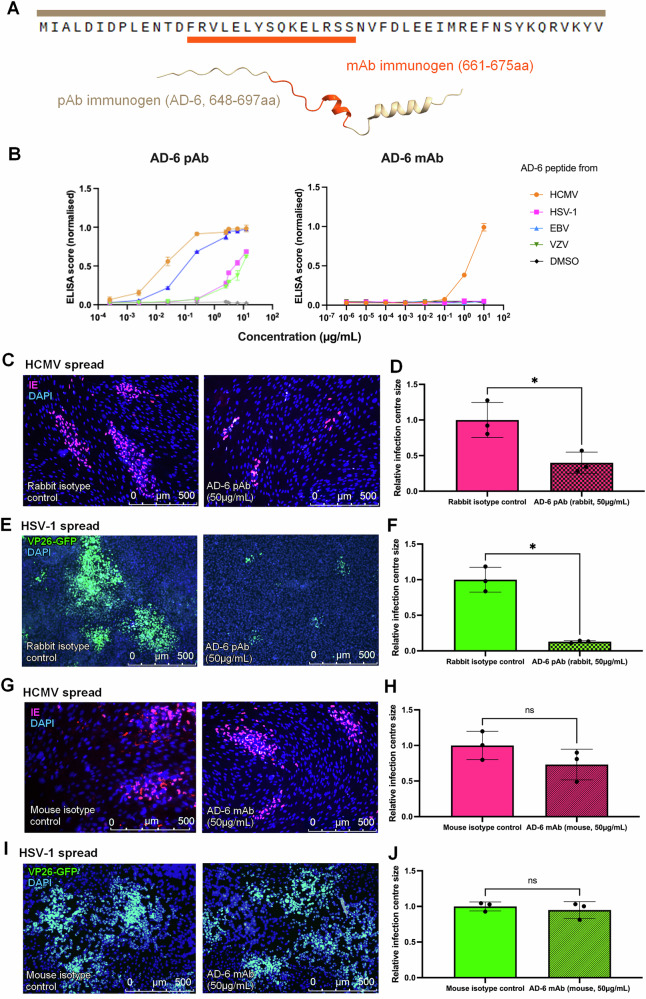
HCMV AD-6 pAb limits the spread of HSV-1. **A** The rabbit anti-AD-6 pAb was generated against the whole-length peptide of AD-6 (highlighted in beige), which forms a structure with potentially linear and non-linear epitopes. The murine anti-AD-6 mAb were generated against a short linear segment of AD-6 (highlighted in orange). **B** ELISA assay measuring the ability of the pAb and mAb to bind HHV AD-6 analogues coated onto plates across a serial dilution. Each dot represents an average between two biological replicates; the error bars represent ±SD. The maximum score of each assay was set as 1 for normalisation. **C**–**J** Cells infected at a low MOI (0.05) with a cell-associated strain of HCMV (**C**, **G**) or HSV-1 (**E**, **I**) were incubated with AD-6 pAb, mAb, or non-immune control and then stained for IE expression (HCMV) or visualised by expression of VP26-GFP (HSV-1). Each dot in (**D**, **F**, **H**, **J**) represents a biological replicate, whereby the size of the infection cluster per well was determined as an average area of at least 10 clusters. Welch’s *t*-test was performed to determine the levels of statistical significance. Data represented as mean ± SD. *p < 0.05; **p < 0.01; ***p < 0.001; ****p < 0.0001, ns non-significant.

To investigate the hypothesis that there are conserved conformational epitopes across different AD-6 analogues, we turned to in vitro assays of AD-6 antibody binding and function. To do this, we utilised our polyclonal AD-6 antibody, which recognises multiple epitopes within HCMV AD-6 compared to a monoclonal AD-6 antibody with much more limited reactivity against epitopes within AD-6 (Supplementary Fig. 5A). First, we measured the ability of HCMV AD-6 pAb and mAb which were raised against different immunogens (Fig. 3A) to bind to other HHV AD-6 polypeptides (Fig. 3B). By simple ELISA, the AD-6 pAb bound all HHV AD-6 polypeptides but, interestingly, the relative binding levels were directly linked to the relationship observed in the phylogenetic tree (Fig. 2E) with binding less strong against the most evolutionarily distant HHV. In contrast, the AD-6 mAb, which was raised against a linear epitope only recognised HCMV AD-6 (Fig. 3B). In addition, a dot blot analysis of native and denatured AD-6 peptides revealed that denaturing EBV and HSV-1 AD-6 polypeptide almost completely abolished binding by the AD-6 antibody pAb whereas it only partially abrogated binding to the full-length HCMV AD-6 polypeptide (Supplementary Fig. 5C). Taken together, these data suggest that the pAb was comprised of antibodies that recognise linear and conformational epitopes and that cross-reactivity may be imparted by conformational recognition.

To look for functional effects, we next investigated whether the AD-6 pAb had anti-viral activity against other HHV. To do this, we assessed the capacity of the pAb and mAb to control HSV-1 cell spread. As expected, the AD-6 pAb dramatically inhibits cell-to-cell spread of HCMV when compared to the rabbit isotype control (Fig. 3C, D). Interestingly, the polyclonal AD-6 antibody also markedly reduced the spread of HSV-1 (Fig. 3E, F). In contrast, the mAb had no significant impact on the spread of either HCMV or HSV (Fig. 3G). Moreover, the AD-6 pAb was also able to potently inhibit the spread (evidenced by the reduction of cytopathic effect) of a model gamma-herpesvirus^23^, HerpesVirus Saimiri (HVS; Supplementary Fig. 6)—further showcasing the cross-herpesvirus anti-viral activity of the HCMV AD-6 pAb.

### Sera from HCMV gB/MF59 vaccinees recognise HSV-1 AD-6

As the study evolved, we considered that a potential paradox of these investigations into conformational epitopes was that AD-6 was originally identified in human sera using peptide mapping against small 15aa peptides^13^, which would, presumably, largely quantify responses against linear epitopes in the gB/MF59 immunogen. Consequently, we decided to investigate whether the same level of HCMV/HSV-1 cross-reactivity against AD-6 peptides could be observed in human sera from HCMV-seronegative recipients of gB/MF59 vaccine, as observed with the rabbit polyclonal antibody.

**Fig. 4:**
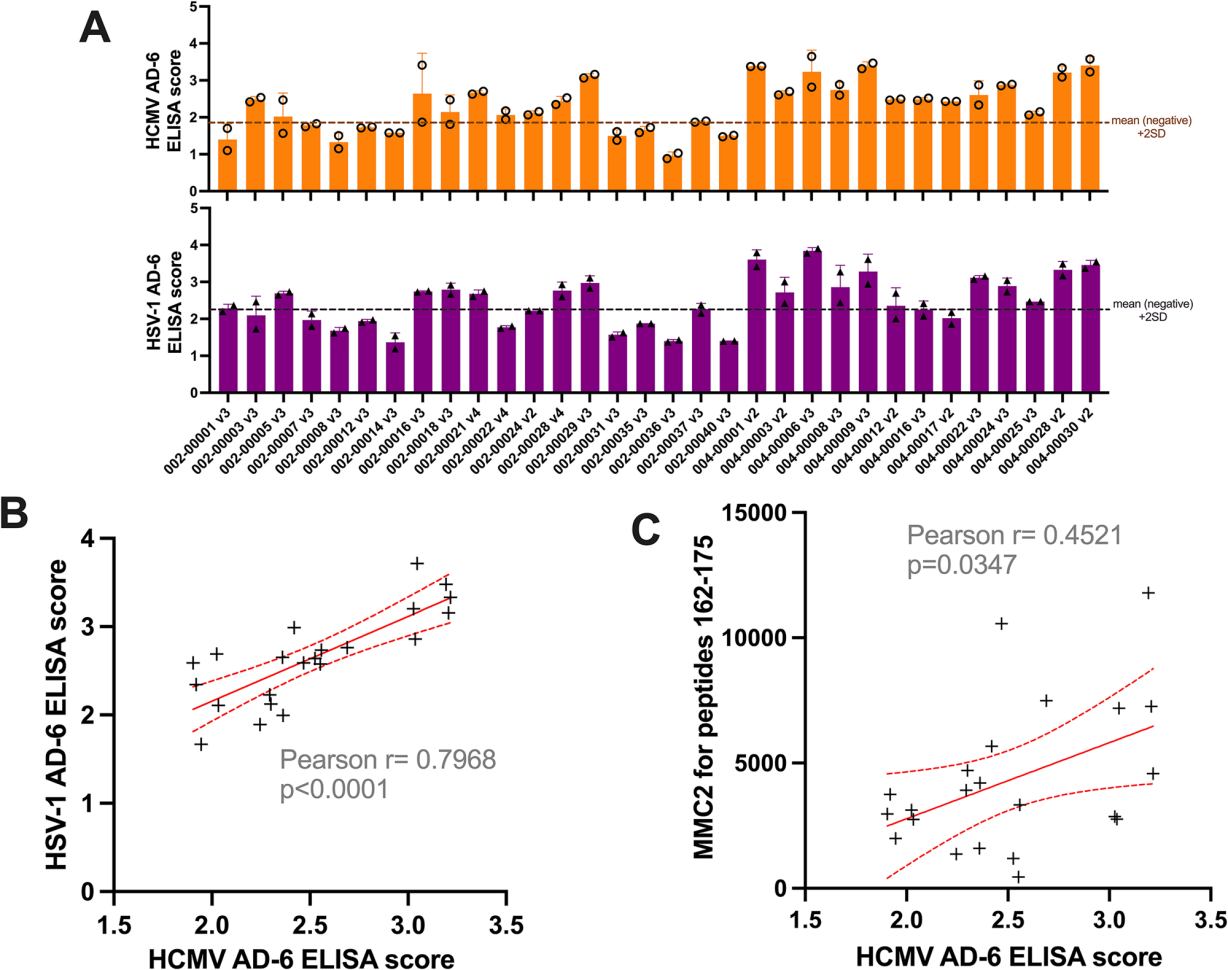
In gB/MF59 vaccine recipients, the HCMV AD-6 and HSV-1 AD-6 responses correlate. **A** ELISA was performed against the HCMV AD-6 and HSV-1 AD-6 peptides using sera from 32 vaccine recipients, and 12 negative samples that included: 7 placebo recipients, 4 seronegative trial participants before vaccine/placebo administration, and 1 healthy seronegative donor. All sera were used at a 1:50 dilution. A cut-off value for HCMV AD-6 response via this assay was calculated as a mean value of the negative samples’ scores plus 2 standard deviations of the negative scores’ distribution and used to identify ‘HCMV AD-6 responders’ for further analyses. **B** The ELISA OD450nm reading for AD-6 responders was plotted for HCMV AD-6 and HSV AD-6 and a correlation analysis was performed. **C** The ELISA OD450nm reading for the HCMV AD-6 ELISA score was plotted against the MMC2 score, used to represent the level of binding against the linear peptides comprising AD-6 identified previously^13^. For both (**B**, **C**), the solid red line represents a simple linear regression fit through the data, and the dashed line represents the 95% CI of the model. The Pearson *r* and the associated *p* value were obtained from Pearson correlation analyses.

Using an ELISA approach, the vaccinee sera recognised both HCMV and HSV AD-6 with a clear direct correlation between the levels of binding HCMV AD-6 and HSV-1 AD-6 peptides for each serum sample (Fig. 4A, B). In contrast, when the average response against peptides (linear) versus total AD-6 (linear plus conformational) was compared for HCMV it was clear that a higher AD-6 ELISA binding score did not necessarily correlate with a higher peptide binding (MMC2) score (Fig. 4C). Taken together these data argue that a component of the response in humans to vaccine gB comprises conformation-specific antibodies and that this was not a phenomenon specific to the rabbit polyclonal AD-6 antibody.

### AD-6 immune domain overlaps with a structural domain of gB displaying high molecular frustration

**Fig. 5:**
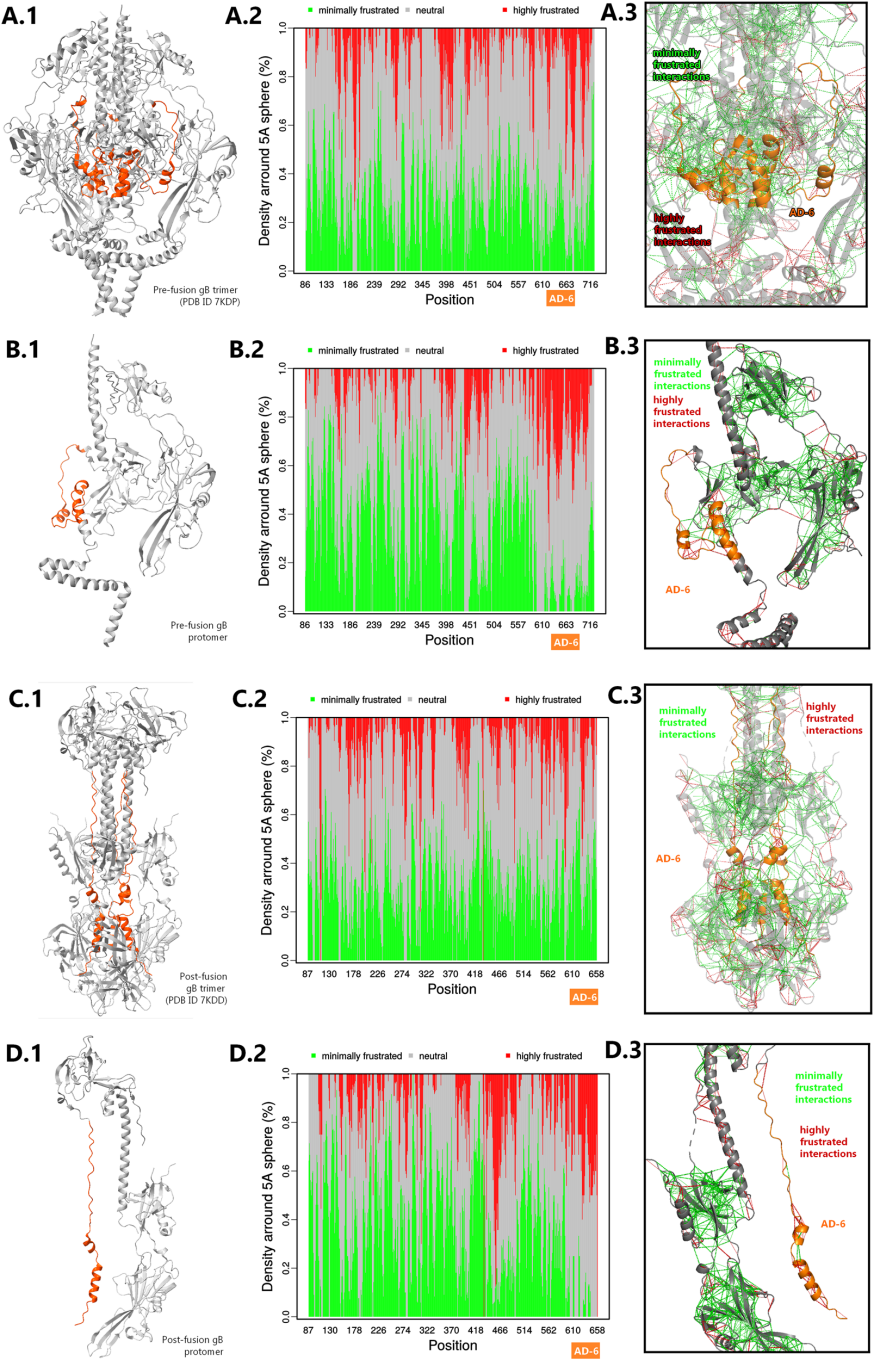
AD-6 maps onto a highly frustrated region of the gB protein. Molecular frustration (MF) analysis plots for pre- (7KDP; **A**, **B**) and post-fusion (7KDD; **C**, **D**) gB in the trimeric (**A**, **C**) and monomeric form (**B**, **D**). AD-6 is highlighted in orange. The A.2, B.2, C.2, D.2 plots’ *X*-axis represents the amino acid sequence of the structure analysed, not accounting for the coordinates of the missing structure. The location of AD-6 on each of the plots was determined by calculating the missing ‘length’ in both pre-fusion and post-fusion structures and adjusting the needed *X*-axis coordinate accordingly. Red highlights areas of high MF, green—areas of minimal MF, grey—neutral areas. Configurational molecular frustration profiles (A.3, B.3, C.3, D.3) obtained from the frustratometer server (frustratometer.qb.fcen.uba.ar).

Finally, we returned to our in silico interrogation of gB to better understand why antibodies directed against conformational epitopes within AD-6 could be mechanistically important for anti-viral activity. To do this, we investigated the physicochemical properties of total gB and AD-6 in the context of gB structure. From a biochemical point of view, polyproteins most often adopt structures that represent the lowest energy state. However, protein function is often dependent on adopting conformations that are energetically less favourable—creating a conflict in the protein^24^—but then can become a source of stored energy. One class of proteins where this is apparent are viral fusion proteins. Fusion is an energy-dependent process which can be stored within the protein and identified by the application of MF^25^. Thus, we interrogated gB to identify regions of MF within gB. Interestingly, the pre-fusion and post-fusion trimers of gB exist in energetically stable states (Fig. 5A, C, highlighted in green). In direct contrast, gB monomers, where interactions with other protomers are negated, are energetically unfavourable (Fig. 5B, D, highlighted in red). Intriguingly, a similar pattern of MF was not observed when the modified gB used in the gB/MF59 vaccine was analysed, where instead protomers were more energetically stable (Supplementary Fig. 7). A caveat of this analysis was that the structure of the gB/MF59 was predicted with the use of AlphaFold3 due to the lack of experimentally-derived structures, so an equivalent AlphaFold3-generated structure of Merlin gB (GenBank NC_006273) was used for comparison as a control for this.

Thus, a high level of MF was detectable around the location of immunological domain AD-6/ structural Domain V in native gB, which could indicate the functional importance of the region. Taken together, these data strongly suggest AD-6 could be an important immunological target due to the key structural and energetic roles the AD-6 region imparts on gB function.

## Discussion

Glycoprotein B is a class III fusion protein^9^ highly conserved functionally and structurally across all herpesviruses^18–20^, that is considered an important target for vaccination^7^. Our recent follow-up study of a phase II trial of a gB vaccine identified a new antigenic domain of HCMV gB we termed AD-6. Through a combination of in silico and in vitro experimentation, we now demonstrate that AD-6 is an important target to prevent viral reactivation, as well as an important target for immunological control of multiple herpesviruses by humoral immune responses directed against conformationally conserved epitopes within AD-6. Finally, our data suggest that antibodies directed against AD-6 may be important because they target a region of gB that could be energetically critical for the function of gB or its proper processing and formation as the functional trimer. Taken together, these data provide strong immunological and biological support for AD-6 being an important target for the control of herpesvirus infection.

A key element of our study is that, despite a low overall amino acid similarity when aligned, we could use the well-established structural similarity to identify the putative AD-6 regions of HHV, which revealed the distinct possibility for the AD-6 analogue peptides to present conformationally conserved epitopes, which will underpin cross-reactivity against peptides with low sequence identity. We hypothesise that these conformational epitopes are targeted, at least in part, by the AD-6 pAb, explaining the anti-viral activity against HSV-1 and a model gamma herpesvirus HVS. This would also be consistent with the observation that human AD-6 antibodies recognise HSV-1 AD-6. This alone argues that the protective nature of the gB/MF59 vaccine^10–12^ could be linked to conformation-specific antibodies, which, in the case of HCMV, would be working alongside antibodies that recognise linear epitopes. This combined activity could explain why the HCMV AD-6 antibody recognises HCMV AD-6 better and works better against HCMV compared to other HHV. Vaccines against other HHV would utilise cognate AD-6 sequences to ensure both conformation and linear epitope recognising antibodies.

In addition, it was interesting to note that AD-6 rabbit antibodies recognised HCMV AD-6 better than HSV-1 AD-6, whereas in the human gB/MF59 vaccine sera, there was very little difference. Although we cannot rule out the difference is human versus rabbit, we hypothesise that a likely reason is due to the immunogen used. Rabbits were inoculated with AD-6 alone, whereas humans were vaccinated with a recombinant gB protein containing AD-6. Whilst both immunogens likely induced conformation-specific antibodies (based on these data), it is possible that AD-6 polypeptide is partly less structured than AD-6 when presented in the context of the larger gB molecule used in gB/MF59. Teasing this out and generating a better understanding of the relative contributions of linear versus conformational epitopes will be important for designing the next HCMV vaccines.

More generally, it raises the question whether functionally similar regions of other fusion proteins for unrelated viruses able to establish persistent infections with similar growth characteristics may be important immunological targets^26,27^. It is likely that antibodies directed against AD-6 are important because AD-6 is located in a structural region (Domain V) crucial for the function of gB^28^. In support of this suggestion, it was evident that the physicochemical properties like hydrophobicity and electrostatic charge distribution of the four identified HHV AD-6 are similar, as well as their predicted immunogenicity profiles, despite the very limited sequence identity. Taken together, the immune epitope AD-6 of herpesvirus gB is a highly structurally and immunologically conserved domain and is therefore likely to have a conserved role across the viral family.

Although we understand that some AD-6 antibodies block cell-to-cell spread (and now, crucially, reactivation), it remains an important question how AD-6 antibodies bind and neutralise gB in the plasma membrane. One of the possible explanations is that some of the gB is at some point present as a monomer on the plasma membrane of an infected cell, which would reveal AD-6, a domain normally buried within gB trimer. Notably, a similar phenomenon was reported for Vesicular Stomatitis Virus—a virus whose fusogen protein G is the characteristic member of class III fusion proteins^19^—whereby there is an evident equilibrium in which both monomeric and trimeric structures of G are present on the infected cell surface^29,30^. Indeed, our analyses would argue that gB monomer is energetically unfavourable, influencing trimerisation to occur, which may hypothetically be blocked by the AD-6 antibody. Alternatively, HCMV gB likely requires energy for its fusogenic activity, similar to other HHV gB^31^, and thus upon the transition from a pre-fusion to post-fusion state, it may be that the predicted hemi-fusion^32^ state pushes AD-6 into an energetically unfavourable conformation which drives the transition—a transition that may be blocked by binding by AD-6 antibodies. Unfortunately, no confirmed hemi-fusion state gB structure exists to test this hypothesis as of yet, but our data would predict that it is energetically unstable (which may explain the lack of a solved structure).

Our findings again outline the serendipitous value of the immunogen used in the gB/MF59 vaccine. Clearly, this vaccine was able to induce a detectable antibody response to a target within the domain that is not normally exposed in the context of a natural infection sufficiently to promote the same AD-6 immune response^13^. This could be attributed to modifications introduced into the immunogen of gB/MF59, leading to a different epitope presentation, especially that of AD-6^12,33^. Our analysis of the energetic profile and MF of gB/MF59 immunogen further outlined its differences with wild-type gB. In particular, the contrasting lack of a highly frustrated region in Chiron gB monomer and the similarly minimally frustrated state as a protomer and trimer could indicate less efficient trimer formation between Chiron gB protomers, which may have led to the more efficient presentation in AD-6 in the Chiron gB derived vaccine (i.e. gB/MF59). This is also consistent with a hypothesis that AD-6 is biologically important to the function of gB, requiring immunological masking to prevent immune recognition^34,35^. Although gB is the fusogen, it, unlike many other viral fusion proteins, requires an interaction to activate it—in the case of gB, this is with gH/gL containing complexes^36^, which is further regulated by glycoprotein:receptor-mediated events^37^. These interactions likely promote the conformational changes in gB required to drive fusion, including the transient exposure of key regions for function, which may include AD-6 (structural Domain V). This suggests the possibility that millions of years of evolution may have selected a virus able to bury deep within its structure a key region which, if displayed to the immune system, would become an immunological Achilles’ heel. Furthermore, because HCMV is highly immunogenic, it is tempting to speculate that some components of the natural immune response act as ineffectual decoys—a hypothesis historically proposed for AD-1 in gB^38,39^. More generally, our results emphasise the value of the investigation of AD-6 functions and the respective immune response as an example of the importance of ‘hidden’ epitopes in microbiological proteins and, in particular, viral fusion proteins^40^.

Returning to the inhibition of reactivation—the importance of this observation cannot be underestimated. The reactivation of HCMV in the transplant setting (in either the recipient or the incoming donor organ) is crucial for dissemination and disease^1^. It is notable that a number of AD-6 responders in the vaccine recipients never had detectable viraemia^13^. One immediate interpretation of this clinical observation is that AD-6 antibodies may have blocked the initial reactivation event. Given that viral load is the predictor (and driver) of disease, this would dramatically impact clinical management of disease^41–43^—for example, by reducing the need for toxic anti-viral drugs. Furthermore, we do not fully understand the importance of sub-clinical reactivation for the persistence of HCMV in healthy people. We can observe it in circulating dendritic cells^44^, but whether this is important for re-seeding latency is less clear. If it were, then there is the potential for AD-6 antibodies to have a clinically important impact by lowering latent load. Measuring this would require a longitudinal study of vaccine recipients, but could be an important secondary benefit of vaccination. It is also important to stress that the use of AD-6 and AD-2 antibodies here is a tool to understand the contribution of cell-associated versus cell-free spread of HCMV in the context of reactivation, rather than to say AD-6 antibodies block HCMV reactivation, but AD-2 antibodies will not. Theoretically, an antibody directed against any glycoprotein complex (including the recently identified gH:UL116:UL141 complex)^45^ could be effective if that complex plays an important role in cell-associated spread, as has been suggested for some gO neutralising antibodies^37^.

Ultimately, our findings demonstrate the insight that can be obtained from combining immunological, physicochemical and structural studies of an epitope/domain to better understand the functional and clinical importance of an immune response hypothesised to be crucial for the protective activity of the gB/MF59 vaccine in vivo. Consequently, we have identified a novel target for inclusion in future vaccine strategies against HCMV and potentially against other members of the whole herpesvirus family, which is likely linked to conserved biochemical properties imparted by the structure, but not sequence, of HHV gB.

## Methods

### In silico methods

Publicly available PDB structures of post-fusion gB were used for the identification of AD-6 analogues in three additional HHV: 7KDD (HCMV), 3FVC (EBV), 3NWF (HSV-1), and 6VLK (VZV). Putative amino acid coordinates of AD-6 peptide were identified visually after aligning the structures using the ‘Matchmaker’ tool in ChimeraX^46,47^. Confirmatory pairwise structure alignment analyses were performed using FATCAT (Flexible structure AlignmenT by Chaining Aligned fragment pairs allowing Twists)^48^, online server, with the results summarised in Supplementary Table 3 and Supplementary Fig. 3. Both ChimeraX and PyMOL (PyMOL Molecular Graphics System, Version 3.0, Schrödinger, LLC; free open-source version compiled from source) were used for structure visualisation and subsequent analyses.

AlphaFold3 server^49^ was used for predicting the structure of Chiron gB trimer, as presented in Supplementary Fig. 7. Configurational MF index was obtained by submitting the relevant structures as PDB files or PDB ID codes to the frustratometer server (http://frustratometer.qb.fcen.uba.ar/).

An NCBI Protein search was completed with search terms relevant to glycoprotein B of each of the four viruses. The relevant multiple sequence alignments were assembled using MAFFT^50^ and further refined manually by removing incomplete or synthetic sequences. Publicly available PDB structures of post-fusion gB were used for the identification of AD-6 analogues in three additional HHV: 7KDD (HCMV), 3FVC (EBV), 3NWF (HSV-1), and 6VLK (VZV). Maximum likelihood phylogenetic trees were generated using the IQTree online server (http://www.iqtree.org/). Immunogenicity profiles were generated with the use of the IEDB epitope prediction BepiPred 2.0 tool^51^.

### THP-1 reactivation and co-culture with HFF

THP-1 cells (THP-1, TIB-202 ATCC) were infected with HCMV (TB40e strain) at MOI = 1, and the latent virus was reactivated at 5 days post-infection with phorbol 12-myristate 13-acetate (PMA) (50 ng/mL). The stimulated THP-1 cells were then co-cultured with HFF (HFF-1, SCRC-1041 ATCC) for 7 days in the presence of a relevant antibody. After 7 days of co-culture, the HFF monolayer was fixed and immunofluorescently stained for IE as a marker of viral infection.

### HSV-1 and HCMV spread assay

Spread assays for both HCMV and HSV-1 were set up in a similar manner with virus-specific optimisations. First, the cell monolayer was infected with the relevant virus at MOI = 0.01. HFF (HFF-1, SCRC-1041 ATCC) cells were used for HCMV assays, ARPE-19 (ARPE-19, CRL-2302 ATCC) for HSV-1. HCMV strain used for the spread assays was Merlin IE2-GFP strain engineered to spread via a cell-associated route^13,17^, a generous gift from Prof Richard Stanton (Cardiff University). The HSV-1 strain used was a derivative of a KOS strain with a VP26-GFP modification^52^ and is a gift of Richard Milne (UCL)^13,17^. The virus was removed after a short period of time (24 h for HCMV, 6 h for HSV-1) and replaced with the antibody of interest. For HSV-1 assays, an additional layer of semi-viscous 0.15% CMC overlay was introduced to reduce cell-free virus and encourage cell-to-cell spread. In HCMV assays, the antibody dilution was refreshed at 5dpi. HCMV spread was stopped at 10dpi and HSV-1 at 48hpi^13,17^.

### Visualisation and quantification

All assays were fixed with ice-cold ethanol at −20 °C for at least 25 min. All HCMV assays were immunostained with an anti-IE mouse primary antibody (anti-CMV IE antibody, Millipore/Sigma-Aldrich; 1:2000) for 1 h, followed by a goat anti-mouse secondary antibody (AlexaFluor568, Thermo Fisher;1:1000) for 1 h, and all assays were further stained with DAPI at 0.01 mg/mL (concentration, 4′,6-diamidino-2-phenylindole, Sigma-Aldrich) for 1 h. HSV-1 VP26-GFP and HCMV IE2-GFP assays were visualised using the GFP-associated fluorescence, where appropriate. Where appropriate, well montages were generated using the Hermes WiScan system (IDEA Bio-Medical). The number of infection clusters per well was determined by counting manually. The area of an infection cluster was determined in ImageJ^53^ using microscopy images with a known scale. An average infection cluster area per well was determined as the mean area of at least 10 cluster measurements in the well.

### ELISA and dot blot

Nunc Immuno Maxisorp plate (Thermo Scientific) was coated with a peptide of interest at a 1 µg/mL concentration in carbonate-bicarbonate buffer and left at 4 °C overnight. The plate was blocked with 2% FCS in PBS, before introducing the primary antibody or human sera, and a relevant secondary antibody (goat anti-rabbit HRP Invitrogen, goat anti-mouse Novus, goat anti-human HRP SouthernBiotech). TMB substrate and 1 M phosphoric acid were used to develop and stop the chromogen reaction. The results were read using a spectrophotometer at 450 nm wavelength. Human sera were used at a 1:50 dilution. Peptide sequences and the manufacturers responsible for their production can be found in Table S2.

For Dot Blot analyses, 50 ng of AD-6 peptides from HCMV, HSV and EBV were spotted directly (native) or first denatured by heating at 95 °C for 5 min in 2x Laemmli buffer and then spotted onto PVDF filter membrane. Following blocking (TBS-T), the blot was incubated with polyclonal AD-6 antibody (10 μg/ml; 1 h, RT) and then with Goat anti-rabbit-HRP (Thermo Fisher; 1:3000 dilution; 1 h, +4 °C) and then visualised by ECL staining and Chemidoc imaging (BioRad).

### Antibodies

Custom AD-6 rabbit polyclonal antibody was produced by GenScript UK and has been described previously^13^. Briefly, the polyclonal antibody was affinity-purified against AD-6 and then eluted, sterile filtered and stored in the absence of preservatives. A non-immune IgG control was harvested from the same rabbit species and also stored preservative-free. The hybridoma cells producing the AD-6 monoclonal antibody (12B22D5 clone) were a kind gift from Dr Marco Thomas at Erlangen University, Germany (Thomas et al.—manuscript in preparation). The monoclonal antibody was purified from filtered cell-free media using the standard ProteinG Sepharose 4 FastFlow protocol (Cytiva). The presence of an antibody was verified by Western blot, and its concentration was determined using a Nanodrop Lite. Preservative-free mouse IgG was used as an isotype control. Finally, the humanised AD-2 antibody (ITC88) was purchased from Creativelabs.

### Peptides

Custom peptides for ELISA were synthesised by Peptide 2.0 and GenScript UK, with the sequence and manufacturer (Supplementary Table 2).

## Supplementary information

Supplementary Data

## Data Availability

All requests for the primary data should be directed to the corresponding author. All sequence and structural analyses are based on deposited datasets with source references. Specifically, publicly available PDB structures of post-fusion gB were used: 7KDD (HCMV), 3FVC (EBV), 3NWF (HSV-1), and 6VLK (VZV). In addition, for studies of pre-fusion HCMV gB PDB file 7KDP was used. The identification of AD-6 sequences was performed using representative gB sequences for different HHVs: UniProt P13201 (HCMV), P03188 (EBV), P06437 (HSV-1), Q4JR05 (VZV).
