# Supplementary material for: Cross-herpesvirus immunity of the cytomegalovirus gB/MF59 vaccine response
Source: NPJ Vaccines. 2025 Dec 5;10:254. doi: 10.1038/s41541-025-01315-6 (PMC12680769; doi:10.1038/s41541-025-01315-6)
Supplement: Supplementary file 1 — Supplementary Data [file 41541_2025_1315_MOESM1_ESM.pdf]

	HCMV	EBV	HSV-1	VZV
**HCMV**	92.15%	32.97%	27.78%	28.29%
**EBV**	32.97%	100%	29.23%	27.98%
**HSV-1**	27.78%	29.23%	94.91%	48.92%
**VZV**	28.29%	27.98%	48.92%	99.18%

Supplementary Table 1 Percentage identity of the amino acid sequences of full-length gB from four HHV. For cognate comparisons the reported identity is between the two sequences with the highest number of non-identical amino acids. For inter-virus comparisons the percentage identity between representative strain sequences was used (UniProt P13201 (HCMV), P03188 (EBV), P06437 (HSV-1), Q4JR05 (VZV)).

Peptide name	Sequence	Manufacturer
HCMV AD-6	MIALDIDPLENTDFRVLELYSQKELRSSNVFDLEEIMREFNSYKQRVKYV	Peptide 2.0
HCMV AD-6a	MIALDIDPLENTD	GenScript UK
HCMV AD-6b	FRVLELYSQKELRSSN	GenScript UK
HCMV AD-6c	VFDLEEIMREFNSYKQRVKYV	GenScript UK
RhCMV AD-6	MISLDIDPLENTDFKALELYSEDELRSSNVFDLEDIMREFNTYKQRMIHV	Peptide 2.0
MCMV AD-6	LIGLDIEPLENTDFKVLELYSKGELRASNVFSLDEIMREYNSQKQHIRTL	Peptide 2.0
GPCMV AD-6	MIALKTEPLENIDFKVLELYSRDELAQANVFDLESIMREYNYQKKRLDFV	Peptide 2.0
EBV AD-6	ISLNTSLIENIDFASLELYSRDEQRASNVFDLEGIFREYNFQAQNIAGLR	Peptide 2.0
HSV-1 AD-6	IDLNITMLEDHEFVPLEVYTRHEIKDSGLLDYTEVQRRNQLHDLRFADIDTV	Peptide 2.0
VZV AD-6	VDLNLTLLKDREFMPLQVYTRDELRDTGLLDYSEIQRRNQMHSLRFYDIDK	Peptide 2.0

Supplementary Table 2

Sequences and manufacturers of peptides used for ELISA experiments within the manuscript.

Structure 1	Structure 2	FATCAT similarity score	p value	Conclusion
7KDD (HCMV)	3FVC (EBV)	1278.8	<0.05	significantly similar
7KDD (HCMV)	3NWF (HSV-1)	1295.68	<0.05	significantly similar
7KDD (HCMV)	6VLK (VZV)	1318.69	<0.05	significantly similar

Supplementary Table 3

Pairwise structural alignment analysis of HHV gB monomers (7KDD HCMV, 3FVC EBV, 3NWF HSV-1, 6VLK VZV) using FATCAT (Flexible structure AlignmenT by Chaining Aligned fragment pairs allowing Twists) online server.

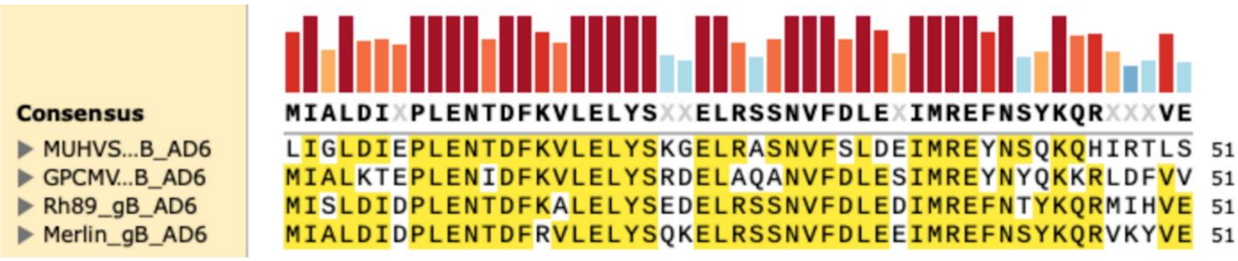

Supplementary Figure 1 AD-6 sequence is conserved within cytomegaloviruses

AD-6 analogues identified within animal CMV gB sequences by direct sequence alignment. MUHVS – mouse CMV (NC_075725.1), GPCMV – guinea pig CMV (NC_020231.1), RhCMV – rhesus macaque CMV (MZ517254.1), Merlin – HCMV (AY446894.2).

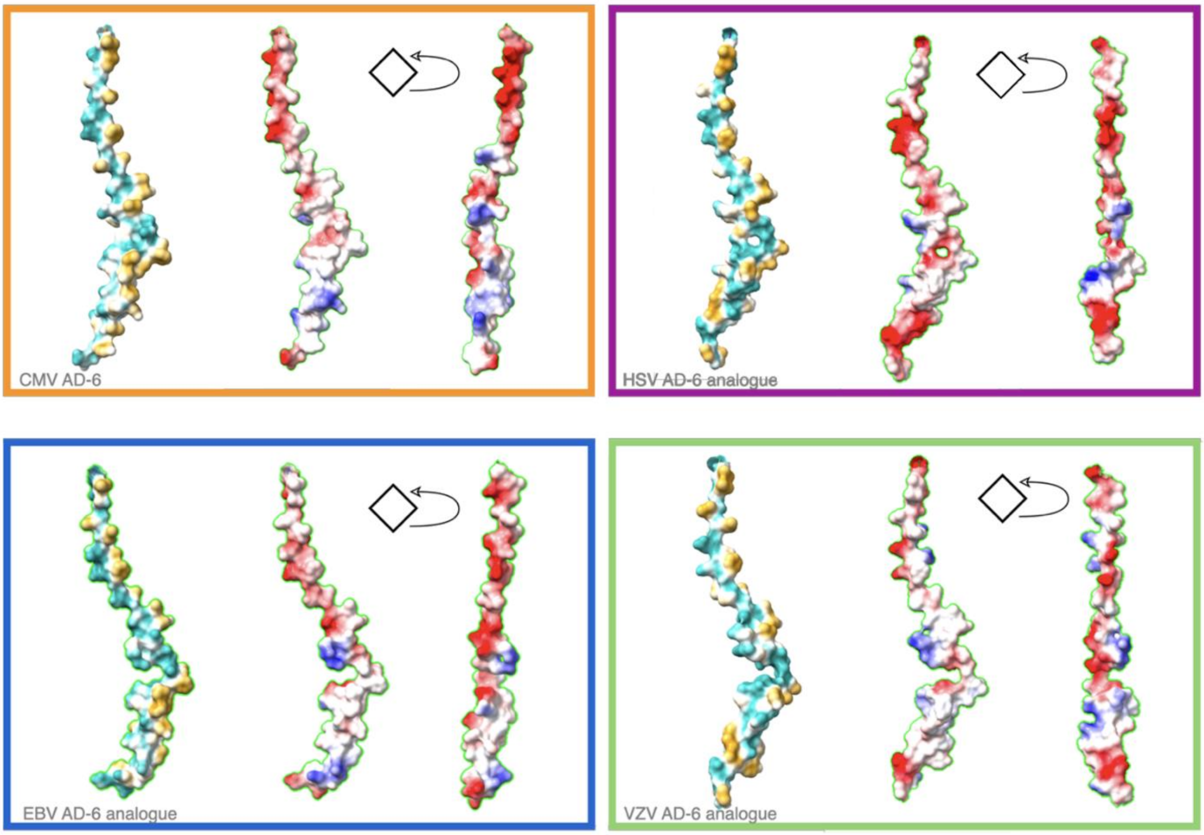

Supplementary Figure 2 AD-6 regions across herpes viruses share physico-chemical properties

Hydrophobicity (teal = hydrophilic, yellow = lipophilic) and electrostatic (red = negative charge, blue = positive charge) of AD-6 was visualised in four HHV gB, using the “Molecule display” option in ChimeraX.

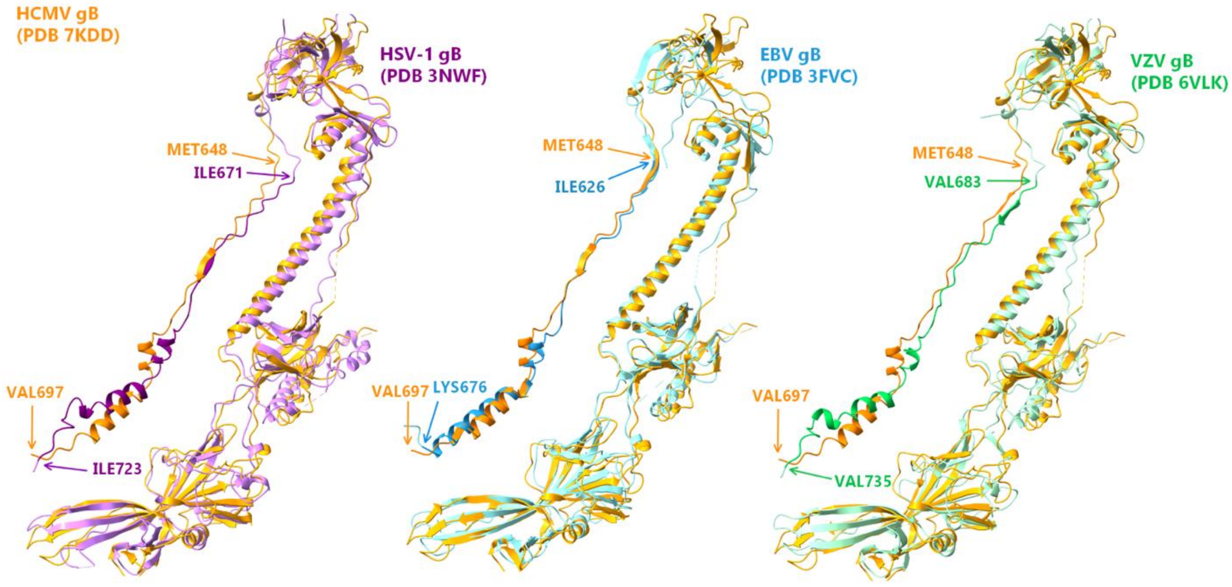

Supplementary Figure 3 Identification of AD-6 using FATCAT alignments

Superimposed structures as aligned by the flexible pairwise methodology of FATCAT (Flexible structure alignment by chaining aligned fragment pairs allowing twists). HCMV post-fusion gB (PDB 7KDD) AD-6 is highlighted in orange. The structural similarity among HHV gB allowed for the identification of putative coordinates of AD-6 analogues in EBV (3FVC), VZV (6VLK), and HSV-1 (3NWF) post-fusion gB monomers.

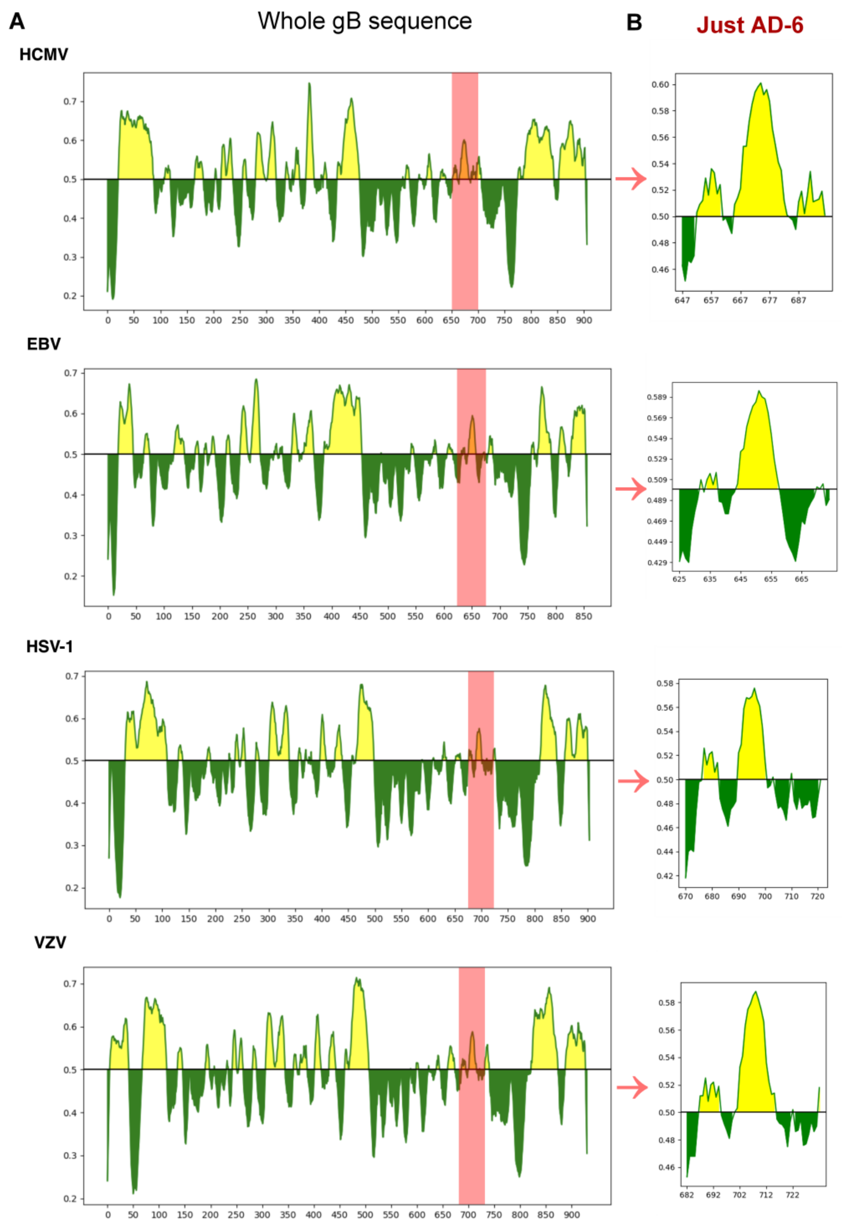

Supplementary Figure 4 AD-6 has similar predicted immunogenicity across the herpes viruses

The gB sequence of HHV were analysed using IEDB B-cell epitope prediction tool to generate immunogenicity plots. Each residue was assigned a predicted immunogenicity score from 0 to 1, with 0.5 used as a cut-off threshold. Whole gB sequence profiles for each virus are presented on the left, with the red shaded block signifying the location of corresponding AD-6 which is then shown in detail on the right.

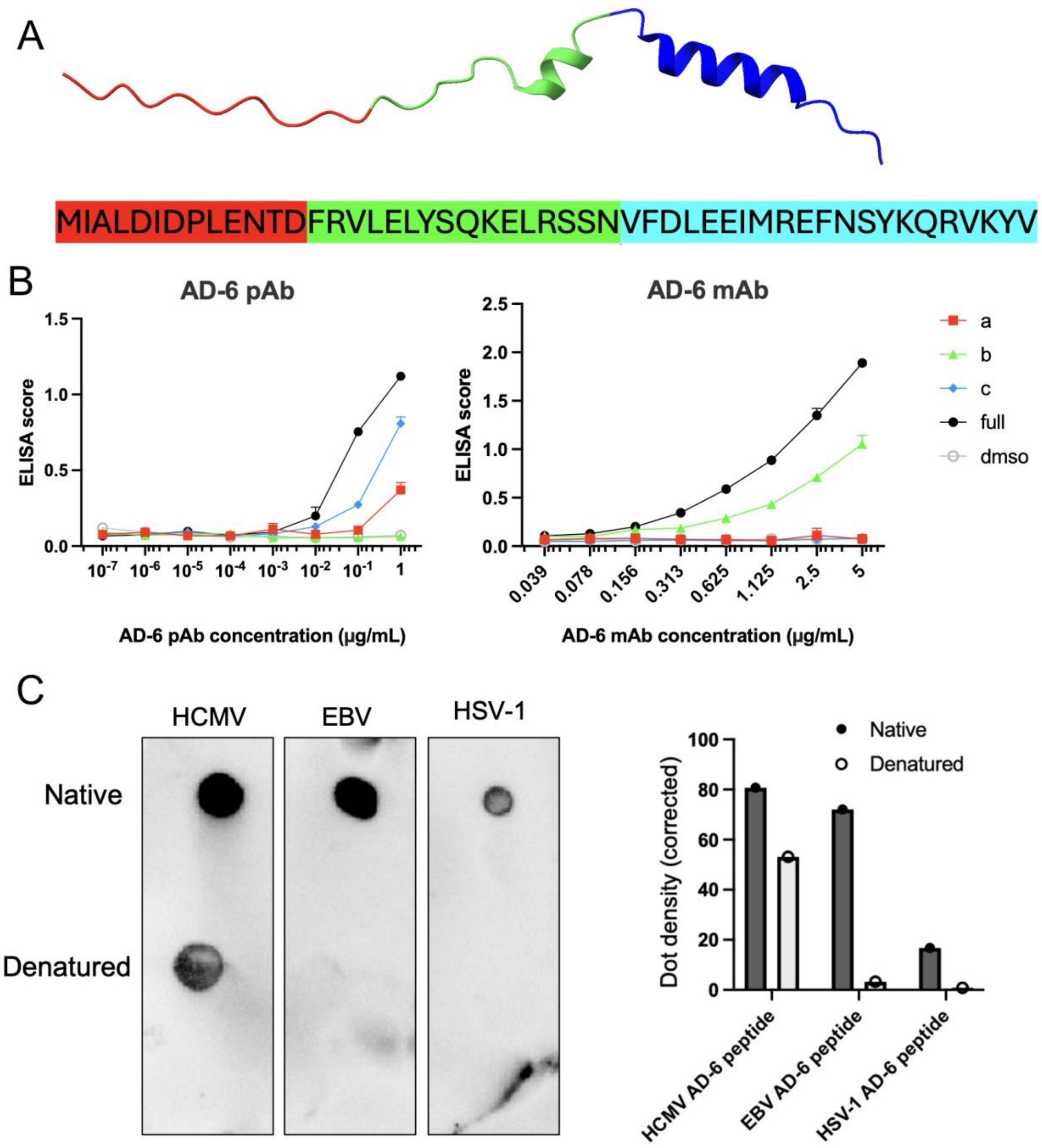

Supplementary Figure 5 Characterisation of AD-6 pAb and AD-6 mAb responses.

(A) AD-6 peptide breakdown into three constituent peptides depending on their secondary structure: AD-6a (disordered region, red), AD-6b (alpha-helix with a disordered tail, green), AD-6c (alpha-helix, blue). (B) AD-6 (black) or short peptides within AD-6 were synthesised and coated onto 96 well plates. ELISA assays were performed by serial dilutions of AD-6 pAb and mAb and binding measured by absorbance at 450nm. (C) A Dotblot spotted with full-length AD-6 peptides from HCMV, EBV, and HSV-1 was performed under native and denaturing conditions with proteins visualised using the AD-6 pAb (10ug/ml) and ECL staining which was analsyed for density using Image J software.

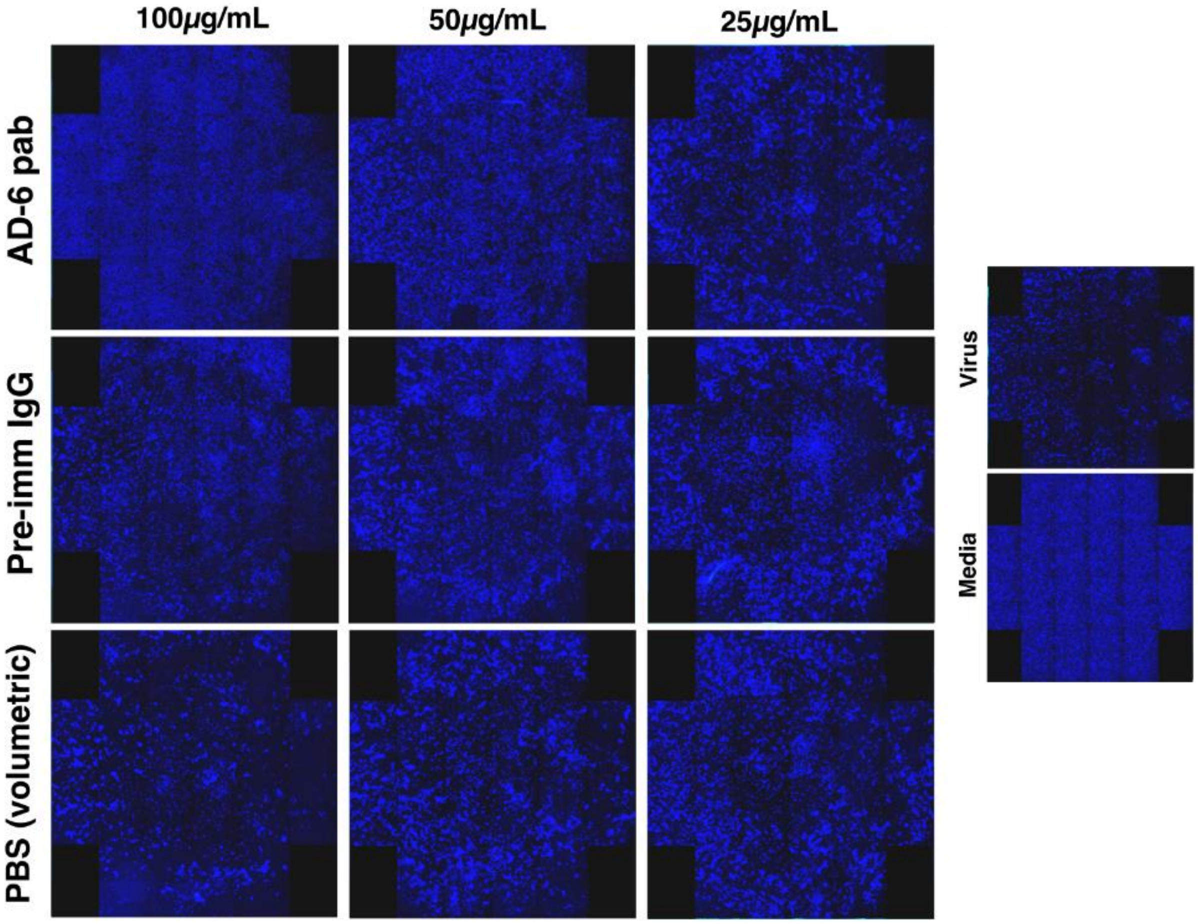

Supplementary Figure 6 AD-6 pAb limits the cytopathic spread of Herpesvirus Saimiri (HVS).

OMK cells were infected with HVS (low MOI) for 2 hours and then cultured with AD-6 pAb, pre-immune rabbit IgG or PBS volumetric control together with 0.15% CMC overlay for 7 days. The pictures presented are montages of 40 microscopy images across the well of a 96 well plate where cells are stained using DAPI to identify nuclei and, indirectly, evidence of CPE.

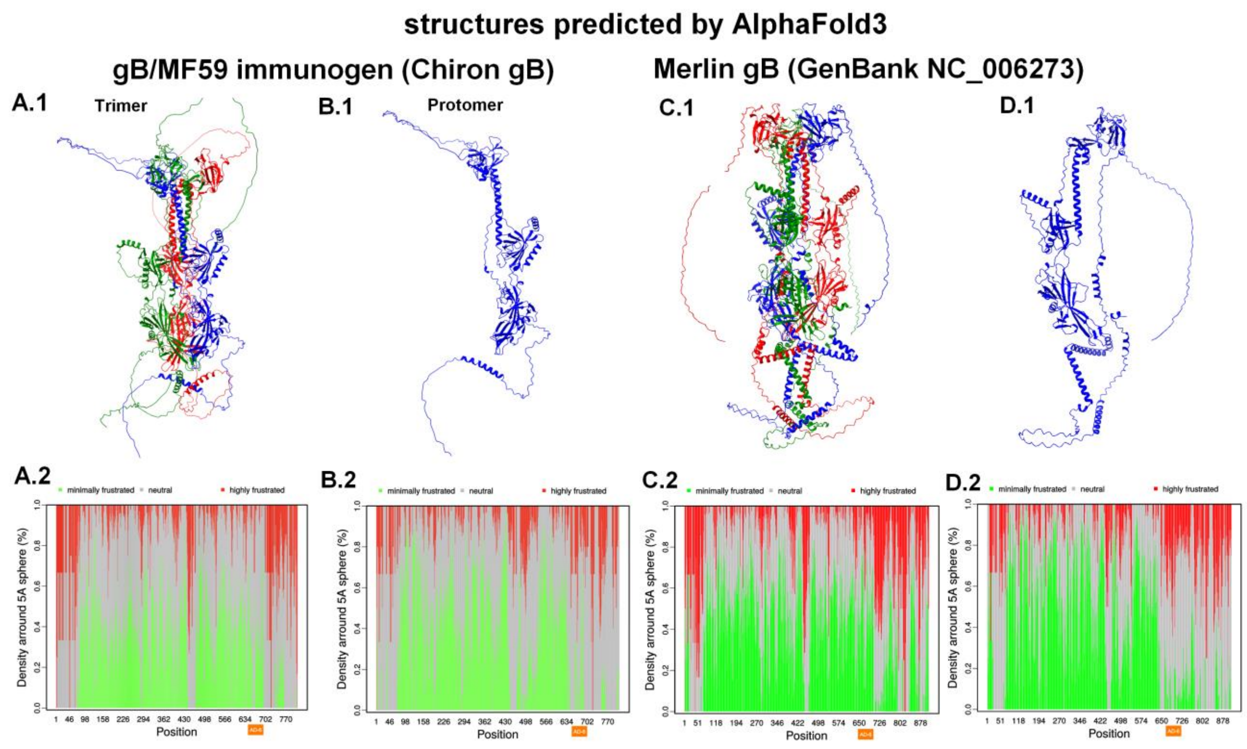

Supplementary Figure 7 AD-6 has reduced molecular frustration in gB/MF59 vaccine

Configurational molecular frustration of the trimer (A) and monomer (B) structures of the immunogen of gB/MF59 vaccine (Chiron gB), as predicted by AlphaFold. Unlike AlphaFold-generated generated protomers of wild-type gB (C,D), the monomer of Chiron gB does not have an area of high molecular frustration around structural domain V (A.2, B.2). MF analysis suggests both trimer and monomer of Chiron gB are largely minimally frustrated proteins. The trimeric structure was obtained via AlphaFold3 in multimer mode. The monomeric structure was obtained by removing other protomers and interactions with them before submission to the frustratometer server.
